# Editorial: Insights in non-neuronal cells: 2021

**DOI:** 10.3389/fncel.2023.1199518

**Published:** 2023-04-26

**Authors:** Jason R. Plemel, Jessica M. Rosin, Marie-Ève Tremblay

**Affiliations:** ^1^Neuroscience and Mental Health Institute, University of Alberta, Edmonton, AB, Canada; ^2^Division of Neurology, Department of Medicine, University of Alberta, Edmonton, AB, Canada; ^3^Department of Medical Microbiology and Immunology, University of Alberta, Edmonton, AB, Canada; ^4^Department of Oral Biological and Medical Sciences, University of British Columbia, Vancouver, BC, Canada; ^5^Life Sciences Institute, University of British Columbia, Vancouver, BC, Canada; ^6^Division of Medical Sciences, University of Victoria, Victoria, BC, Canada; ^7^Institute on Aging and Lifelong Health, University of Victoria, Victoria, BC, Canada; ^8^Center for Advanced Materials and Related Technology, University of Victoria, Victoria, BC, Canada; ^9^Neurosciences Axis, Centre de Recherche du CHU de Québec, Université Laval, Québec City, QC, Canada; ^10^Department of Molecular Medicine, Université Laval, Québec City, QC, Canada; ^11^Department of Neurology and Neurosurgery, McGill University, Montreal, QC, Canada; ^12^Department of Biochemistry and Molecular Biology, University of British Columbia, Vancouver, BC, Canada

**Keywords:** astrocytes, microglia, pericytes, endothelial cells, oligodendrocytes, mouse, ferret, human

We are now entering the third decade of the 21st Century, and, especially in the last years, the achievements made by scientists have been exceptional, leading to major advancements in the fast-growing field of non-neuronal cells, including resident immune cells, glial cells (astrocytes and cells of the oligodendrocyte lineage), neurovascular cells (endothelial cells, pericytes, perivascular macrophages, etc.), and other cells that populate the nervous system.

Frontiers has organized a series of Research Topics to highlight the latest advancements in science in order to be at the forefront of science in different fields of research. This editorial initiative is focused on new insights, novel developments, current challenges, latest discoveries, recent advances, and future perspectives in the field of non-neuronal cells.

The goal of this special edition Research Topic was to shed light on the progress made in the past decade in the non-neuronal cells field and on its future challenges to provide a thorough overview of the status of the art. This article collection, which aims to inspire, inform, and provide direction and guidance to researchers in the field, comprises seven articles.

Two Brief Research Reports focus on the development of tools allowing to study the roles of astrocytes and microglia across various contexts of health and disease. Kim et al. first describe the generation and characterization of a conditional transgenic mouse model in which lipocalin-2 (LCN2), an adipokine associated with inflammation, is expressed in a cell type manner. With this model, the authors successfully induced the expression of LCN2 in astrocytes. This resulted in increased hippocampal inflammation and glial reactivity, as well as cognitive impairment.

Ogaki et al. present the development of an *ex vivo* transplantation protocol in which human induced pluripotent stem cell-derived microglia (hiPSC-MG) are transplanted into cultured mouse hippocampal slices. This novel protocol allowed to replace ~80% of the endogenous microglial population. When neuronal death was induced using kainate, these hiPSC-MG were reactive, changing their morphology and increasing their phagocytosis.

Three Original Research articles next provide novel insights into the implication of pericytes in pathology, the changes in astrocytes across evolution, and their role in experience-dependent homeostatic plasticity. Choe et al. studied in mice the outcomes of pericyte depletion on capillary stalling, a transient interruption of microcirculation associated with a wide variety of neurological diseases. Their findings identify increased interactions between leukocytes and endothelial cells as a key mechanism underlying this capillary stalling.

Roboon et al. examined the features of astrocytes in ferrets—whose brains show similarities to humans—by isolating these cells from the neonatal brain. Ferret astrocytes were cultured, then their morphology, calcium response, proliferating ability, and gene expression, were compared with those of mouse or human astrocytes. This investigation revealed that ferret astrocytes present different properties compared to mouse astrocytes, and similarities in gene expression with human astrocytes, suggesting ferret astrocytes may help explain brain evolution.

Butcher et al. investigated the roles of astrocytic calcium signaling during experience-dependent plasticity by determining the outcomes of complete whisker ablation in mutant mice (IP_3_R2^−/−^) in which astrocytes are lacking the IP_3_ receptor. The resulting changes required astrocytic IP_3_ signaling, as they were not observed in the mutants. Experiments with whisker stimulation also supported involvement of the astrocytic IP_3_ receptor during long-term depression, suggesting that several mechanisms of experience-dependent plasticity require astrocytic calcium signaling.

The Research Topic further presents a Hypothesis and Theory article and a Review article which together shed light onto the neuroglial mechanisms behind chronic fatigue syndrome/myalgic encephalomyelitis (ME/CSF) and COVID-19. Renz-Polster et al. propose the hypothesis that a common denominator of pathobiological processes in ME/CFS may be central nervous system dysfunction due to impaired or pathologically reactive neuroglia (astrocytes, microglia, and oligodendrocytes). Key features of ME/CSF are also recognized in a subset of patients with post-acute sequelae COVID-19, making the discussed mechanisms pertinent to this entity. Gonçalves et al. cover the role of astrocytic calcium-binding proteins. Astrocytes are key to integrating energy metabolism with neurotransmission and brain inflammation. The discussed mechanisms are compromised in pathological conditions, notably COVID-19. Potential neuroprotective targets, notably focused on the restoration of calcium homeostasis, are identified.

## Author's note

M-ÈT is a Tier 2 CRC in Neurobiology of Aging and Cognition. JP is a Tier 2 Canada Research Chair in Glial Neuroimmunology. JR is a Tier 2 Canada Research Chair in Immune Regulation of Developmental Programs.

## Author contributions

M-ÈT drafted the editorial and which was revised by JP and JR. All authors contributed to the article and approved the submitted version.

